# Viral Carcinogenesis: Factors Inducing DNA Damage and Virus Integration

**DOI:** 10.3390/cancers6042155

**Published:** 2014-10-22

**Authors:** Yan Chen, Vonetta Williams, Maria Filippova, Valery Filippov, Penelope Duerksen-Hughes

**Affiliations:** Department of Basic Science, Loma Linda University, Loma Linda, CA 92354, USA; E-Mails: yachen@llu.edu (Y.C.); vwilliams@llu.edu (V.W.); mfilippova@llu.edu (M.F.); vfilippov@llu.edu (V.F.)

**Keywords:** virus, DNA damage, ROS, carcinogenesis, HBV, HPV, MCV, EBV, integration

## Abstract

Viruses are the causative agents of 10%–15% of human cancers worldwide. The most common outcome for virus-induced reprogramming is genomic instability, including accumulation of mutations, aberrations and DNA damage. Although each virus has its own specific mechanism for promoting carcinogenesis, the majority of DNA oncogenic viruses encode oncogenes that transform infected cells, frequently by targeting p53 and pRB. In addition, integration of viral DNA into the human genome can also play an important role in promoting tumor development for several viruses, including HBV and HPV. Because viral integration requires the breakage of both the viral and the host DNA, the integration rate is believed to be linked to the levels of DNA damage. DNA damage can be caused by both endogenous and exogenous factors, including inflammation induced by either the virus itself or by co-infections with other agents, environmental agents and other factors. Typically, cancer develops years to decades following the initial infection. A better understanding of virus-mediated carcinogenesis, the networking of pathways involved in transformation and the relevant risk factors, particularly in those cases where tumorigenesis proceeds by way of virus integration, will help to suggest prophylactic and therapeutic strategies to reduce the risk of virus-mediated cancer.

## 1. Introduction

Viruses are the causative agents of approximately 10%–15% of all cancers worldwide. Viruses that have been linked to carcinogenesis include several DNA viruses: Kaposi’s sarcoma herpesvirus (KSHV), Merkel cell polyomavirus (MCV), Epstein-Barr virus (EBV), Human papillomavirus (HPV), hepatitis B virus (HBV) and the simian virus 40 (SV40), as well as at least two RNA viruses: human T-lymphotropic virus-1 (HTLV-1) and the hepatitis C virus (HCV). These viruses and their associated cancers are shown in Table 1.

The mechanism of virus transmission varies depending on the type of virus, the type of infection, its targets and the associated life cycle. Sexual transmission has been attributed to HBV [1], HCV [2] and HPV [3]. HBV and HCV infections can be also acquired from contaminated blood transfusions [4,5] or can be transmitted through needle sharing between intravenous drug users [5,6]. Perinatal transmission of HBV [7] and HCV [8] has been observed from mother to child during pregnancy or labor, and EBV infections can be transmitted through saliva from one individual to another [9]. SV40 has the potential to be introduced into humans by the poliovirus vaccine [10].

Progression to cancer as a result of infection with an oncogenic virus is usually a rare event. For example, the overall prevalence of high-risk HPV infection is 23% [11]. Most of these infections clear up without any intervention within a few months after acquisition, and about 90% clear within two years. Only 0.3%–1.2% of these initial infections will eventually progress to invasive cervical cancer, according to 2013 World Health Organization (WHO) data [12]. Another example relates to the hepatitis B virus (HBV). The highest rate of infection was found in Sub-Saharan Africa and East Asia, where the prevalence of chronic hepatitis in the adult population is between 5%–10% according to 2014 WHO data [13]. More than 90% of those infected people will recover and be completely cleared of the virus within six months, while less than 5% of infected people will develop chronic hepatitis. Twenty percent of chronic hepatitis B cases will progress to hepatic cirrhosis, and of these, only 5% will progress to hepatocellular carcinoma (HCC). In the case of another agent causing HCC, HCV, approximately 2%–3% (130–170 million) of the worldwide population has been infected with HCV [14]. According to 2014 data from the WHO, chronic HCV infection will develop in 55%–85% of infected persons, with the risk of hepatic cirrhosis being approximately 15%–30% within 20 years [15]. The transformation rate of cirrhosis to HCC is approximately 1%–3% per year [5,16]. According to WHO serological testing data, EBV, another oncogenic virus, is present in approximately 95% of adults worldwide, including those residing in the United States. However, only in very rare cases do these infections progress to Burkitt’s lymphoma or nasopharyngeal carcinoma. Similarly, the prevalence of HTLV-I infection in some endemic areas is 15% [17], while adult T-cell leukemia (ATL) only develops in an estimated 2%–4% of these infected persons in endemic regions, and only where early childhood infection is common [18,19]. Overall, these data indicate that infection with oncogenic viruses does not mean the obligatory development of cancer, although infected individuals can be considered an at-risk population for cancer development. Epidemiological studies demonstrate that risk factors for cancer include virus load, persistence of infection and duration of infection [20,21].

Although these viruses belong to different groups, display different etiologies, carry out a variety of life cycles, target different organs and utilize variable mechanisms to induce cancer, some common features of cancer development can be noted. Overall, these common features are related to virus-mediated and exogenously-derived factors that create favorable conditions for cancer promotion and progression. In this current review, we summarize data on the factors that induce alterations in host genomes that contribute in tumorigenesis. We will also discuss the outcome of DNA damage on viral genome integration, which plays a pivotal role in cancers induced by some viruses.

**Table 1:** Oncogenic viruses and associated cancers. HTLV-1, human T-lymphotropic virus-1; KSHV, Kaposi’s sarcoma herpesvirus; SV40, simian virus 40.

Virus	Genome	Associated Cancers
HTLV-I	Positive-strand, single-stranded RNA retrovirus	Adult T-cell leukemia (ATL) [22]. ATL is a malignancy of CD4+ T-lymphocytes, which exhibits severe immunodeficiency and resistance to intensive chemotherapies [23].
HCV	Positive-strand, single-stranded RNA flavivirus	Some hepatocellular carcinoma (HCC) and possibly some lymphomas [24,25]. The risk of HCC is 11.5- to 17-fold in HCV-infected patients [26,27].
KSHV	Double-stranded DNA herpesvirus	Kaposi’s sarcoma, primary effusion lymphoma [28]. Kaposi’s sarcoma is the most frequent cause of malignancy among AIDS patients.
MCV	Double-stranded DNA polyomavirus	Merkel cell carcinoma (MCC) [29]. MCC is a rare, but aggressive human skin cancer, and it typically affects the elderly, as well as immunosuppressed individuals.
EBV	Double-stranded DNA herpesvirus	Most Burkitt’s lymphoma and nasopharyngeal carcinoma [30].
HBV	Partially double-stranded DNA hepadnavirus with retroviral features	Chronic infection with HBV has been linked to the development of HCC for over 30 years [31,32].
HPVs	A group of circular, double-stranded DNA viruses [33].	High-risk human papillomaviruses (HPV) 16 and HPV 18 (some other α-HPV types are also carcinogenic) are associated with cervical cancer, penile cancers and some other anogenital and head and neck cancers [34,35].
SV40	Polyomavirus of the rhesus macaque [10]	SV40 sequences are detected in 60% of human mesothelioma, a rare tumor related to exposure to asbestos [36]. It is also detected in brain tumors [37,38,39], osteosarcoma [40] and non-Hodgkin lymphoma (NHL) [41,42].

## 2. Mechanisms of Viral Carcinogenesis

Cellular transformation is a multi-step process that results in the transformation of healthy cells into cancer cells. It requires a progression of changes at the cellular, genetic and epigenetic levels that ultimately lead to the cellular changes necessary for uncontrolled cell division and formation of a malignant mass. Hanahan and Weinberg, in their landmark review “Hallmarks of Cancer”, listed six essential alterations that must occur in a cell’s physiology to cause malignancy, including self-sufficiency in growth signals, insensitivity to growth-inhibitory (antigrowth) signals, evasion of programmed cell death (apoptosis), limitless replicative potential, sustained angiogenesis and tissue invasion and metastasis [43,44]. More recently, two emerging hallmarks have been added to the list: deregulating cellular energetics and avoiding immune destruction [44]. Cellular transformation induced by oncogenic viruses also adheres to Weinberg’s hallmarks; in particular, limitless replicative potential, evasion of apoptosis and genome instability [43]. Viruses, when functioning as carcinogenic agents, utilize a variety of carcinogenic mechanisms to transform human cells. One such mechanism is direct transformation, where the virus expresses viral oncogenes that can directly transform infected cells. Several viruses, including HPV, EBV, KSHV, SV40, HCV and HTLV, encode oncoproteins that employ several mechanisms to inactivate two of the major regulators of genome stability, cell viability and cell cycle; namely, the p53 and retinoblastoma proteins (pRB) [45].

### 2.1. Direct Transformation through Expression of Viral Genes

The tumor suppressor p53 is the product of the TP53 gene; it induces cell cycle arrest or apoptosis in response to cellular damage or insult, guards against genomic instability and plays a critical role in DNA repair [46]. Inactivation of p53 or depletion of its function in infected cells results in an accumulation of genomic mutations and DNA breaks, accumulating genomic instability, a loss of growth suppression and apoptosis, leading to promotion of cellular transformation [46,47]. Perhaps the best-studied example of viral inactivation of p53 is provided by the E6 protein from high-risk (HR) HPV. The HR HPV E6 protein induces ubiquitin-mediated degradation of p53, resulting in disabling of the normal cellular response to many insults, including the DNA damage response [48]. Another example of a virus with the ability to inactivate p53 is KSHV. The large multifunctional protein latency-associated nuclear antigen 1 (LANA1) expressed by KSHV interacts efficiently with p53, represses its transcriptional activity and inhibits p53-induced cell death [49]. Furthermore, the SV40 large T antigen (Tag) is well-known for its ability to bind and inactivate p53 [50]. In addition, it also plays a crucial role in cell-cycle derangement of human mesothelial cells [51], followed by transformation of the cells [52]. In the case of HCV, the NS5A viral oncoprotein is involved in apoptosis inhibition, signal transduction, transcription, transformation and the production of reactive oxygen species (ROS). In particular, NS5A has been shown to bind directly to p53 and to repress transcription of the tumor suppressor p21WAF1 in a p53-dependent manner [53,54,55].

In addition to p53, the tumor suppressor, pRB, is also a frequent target for oncogenic viruses. pRB regulates apoptosis during development, and its loss results in deregulation of growth and apoptosis [56]. In normal cells, pRB is hypophosphorylated in early G1, and becomes increasingly phosphorylated by cyclin D/CDK4/6 complexes as the cell moves towards S phase in response to a signal to divide. This results in the release of the E2F protein, which then activates the transcription of genes required for the S-phase transition [57]. E7, another HPV oncogene, mimics this process by binding to pRB and releasing the E2F protein [58], thereby driving quiescent, infected cells back into a proliferative state in order to enable viral genome replication [59]. In addition, the HR HPV E7 protein binds directly to E2F-1, leading to the activation of E2F-1-dependent transcription, and the affinity of E7 for E2F-1 appears to correlate with the oncogenic potential of the HPV [60]. Therefore, HR HPV E6 works together with E7 to induce cellular genomic instability and mitotic defects, known contributors to carcinogenesis [61]. Another example of pRb inactivation is provided by the large T antigen (Tag) expressed by SV40. One domain of Tag, the LxCxE motif, binds to pRB and inactivates the protein [62,63]. Despite the fact that adenoviruses do not cause cancer in humans, they can disrupt the pRB-E2F interactions via the activity of E1A, thus driving expression of viral transcription and inducing cell cycle progression [64].

In addition to these two major targets, pRB and p53, several other molecules serve as common targets for oncogenic viruses. Telomerase, which is usually found in embryonic cells and is absent in somatic cells, is one such target. In normal cells, the telomeric regions shorten with each round of division [65]; inappropriate expression of telomerase can lead to immortalization. HR HPV E6 can activate the expression of the catalytic subunit of telomerase, telomerase reverse transcriptase (hTERT) [66,67], in order to maintain telomeres through telomerase activation [68,69], and thereby contributing to immortalization. Another virus, EBV, encodes a principal oncoprotein, latent membrane protein 1 (LMP1), which is also able to activate hTERT [70]. HTLV-I expresses Tax protein, which is believed to be critical in the transformation of infected cells to adult T-cell leukemia (ATL). Tax is a 40-kDa trans-regulatory protein encoded by the tax gene located in ORF IV of the pX region [71]. Tax can repress the expression of hTERT by competition with c-Myc through a canonical c-Myc binding site within the hTERT promoter [72]. In addition to this function, it can also reprogram G1 to S progression through direct protein-protein binding, transcriptional induction/repression and post-translational modification [71]. Another target of oncogenic viruses is the tumor suppressor RASSF1A. The inactivation of this gene is correlated with the hypermethylation of its CpG-island promoter region, and the silencing of RASSF1A induces telomerase activity [73]. Tag of SV40 not only inactivates p53, but also inactivates the RASSF1A gene [74,75].

Several other signaling pathways involved in carcinogenesis are directly regulated by multifunctional viral oncoproteins. For example, the SV40 Tag can activate growth factor receptors, such as Met [76], Notch-1 [77] and IGF-1R [78], leading to the activation of the ERK-kinase and AP-1 pathways that promote cell division and contribute to SV40-induced carcinogenesis [79]. In addition, NS5A, the HCV protein that depletes p53, can also interact with Bax and prevent apoptosis in a p53-independent manner [80]. KSHV can express an interferon regulatory factor (IRF)-like signal-transduction protein, ORF K9, and this protein inhibits interferon-induced signaling pathways. This inhibition allows KSHV to overcome interferon-mediated antiviral activity and, thus, can contribute to host cell transformation [81]. The LMP1 oncoprotein expressed by EBV [82], as well as Tax expressed by HTLV-I [23], both target the Nuclear Factor-κB (NF-κB) pathway. The NF-κB pathway plays a critical role in regulating the immune response to infection. NF-κB is a crucial mediator of inflammation-induced tumor growth and progression, as well as an important modulator of tumor surveillance and rejection [83].

### 2.2. Indirect Transformational Activities

In addition to these direct mechanisms underlying virus-induced carcinogenesis, virus-induced chronic infection and inflammation can also function as indirect transforming agents [84]. In fact, Colotta *et al.* in a 2009 paper in *Carcinogenesis* refer to inflammation as the seventh hallmark of cancer [85]. Evidence of the carcinogenic potential of inflammation is provided by chronic inflammatory bowel diseases, such as chronic ulcerative colitis and Crohn’s disease, which can lead to colon cancer, even in the absence of other factors [86]. Another well-known example is the chronic infection and inflammation induced by *Helicobacter pylori*, which results in stomach cancer worldwide [87]. Inflammation can also be caused by infection with viruses, thereby providing another linkage between infection and carcinogenesis. For example, chronic HBV or HCV infection can lead to hepatocellular carcinoma (HCC) through a process that induces cell death, regeneration, cirrhosis and, finally, cancer [55,88,89]. In many cases, the inflammation induced by chronic infection creates a microenvironment that favors expression of viral oncogenes. For example, in HBV-induced HCC, HBV is clonally integrated into host DNA, and the integrated HBV sequences encode HBV X (HBx) and/or truncated envelope pre-S2/S proteins in a large portion of the HCC. These oncoproteins are thought to participate in directly promoting transformation of hepatocytes to HCC [55]. Another example of the way in which virus infections can indirectly contribute to carcinogenesis is provided by cutaneous papillomavirus infections, which contribute indirectly to skin carcinogenesis by blocking apoptosis in cells exposed to ultraviolet light and, thus, permitting the survival of UV-damaged cells [90].

## 3. DNA Damage and Viruses

p53 has been termed “the guardian of genome integrity.” Its depletion or inactivation by virus proteins, as well as the inflammation produced as a result of chronic infection, both lead to an accumulation of point mutations, genomic instability and DNA damage. DNA damage starts with a chemical modification to a base of DNA that induces a break in either one or both strands of the DNA [91]. In uninfected cells, DNA repair systems can recognize DNA damaged bases as abnormal structures and repair the damage prior to replication. The cell can call on several systems to repair DNA damage caused by both endogenous and exogenous factors, and these DNA repair pathways recognize both single-strand breaks (SSB) and double-strand breaks (DSB). SSB DNA repair mechanisms include direct repair, nucleotide excision repair, mismatch repair and base excision repair. The DSB repair mechanisms include homologous recombination and nonhomologous end joining [92]. If the DNA damage cannot be completely repaired, this damage can lead to a deregulated cell cycle, genomic instability [47] and mutations associated with the development of cancer [93,94]. However, activation of DNA damage response and cell cycle regulation by virus proteins benefit virus production by providing an S-phase-like replication environment, preventing apoptosis and promoting episome maintenance [47].

### DNA Damage and Virus Infection

During viral infections, the host must maintain genome integrity through the activation of its surveillance network for detecting and repairing DNA damage. Many viruses can employ direct and/or indirect mechanisms to activate DNA damage signaling pathways [47], and this DNA damage signaling can be activated either by virus infection itself or the expression of viral proteins. One example of DNA damage signaling activated by virus infection is seen in the case of EBV infections, where infection induces the cellular DNA damage response and activates the ataxia telangiectasia-mutated (ATM) signal transduction pathway [95]. ATM is the primary signal kinase activated after sensor proteins detect DNA damage. Autophosphorylation of ATM can activate downstream substrates, such as checkpoint kinase Chk2 and the DNA damage response protein, 53BP1. Inhibition of ATM and Chk2 significantly increases the transformation efficiency of EBV-infected primary B-cells [96]. Another example is seen in the case of SV40 infection, where infection results in activation of ATM and endogenous ATM substrates [97]. SV40 viral proteins can also induce the DNA damage response, as expression of the SV40 Tag protein activates the DNA damage response via binding to the mitotic spindle checkpoint kinase, Bub 1 [98]. Activation of the DNA damage response is in the best interest of the virus, as it promotes SV40 viral DNA replication [97]. In the case of HPV, expression of viral proteins from the HPV episome leads to ATM kinase activation, which, in turn, activates Chk2. Caspase activation due to Chk2 activity is necessary for productive viral genome amplification [99,100]. In any case, if the DNA damage repair system activated by the virus is not sufficiently efficient or if additional DNA damage induced by exogenous sources accumulates in the host genome, one side effect of this increased DNA damage in infected cells is the ability of viral genomes to become integrated into the host genome. In the case of HPV and HBV, viral genome integration is a major trigger point for the development of cancer. The role and importance of viral genome integration in cancer development will be discussed below.

## 4. Factors that Cause DNA Damage and that Contribute to Carcinogenesis

Factors that induce DNA damage in infected cells can be divided into endogenous, *i.e.*, virus mediated, and exogenous factors.

### 4.1. Inflammation and DNA Damage Induced by Virus Itself

Inflammation is a primary immune response to infection by pathogens [101]. That process involves activation and directed migration of leukocytes from the venous system to the sites of infection; tissue mast cells also play a significant role [86]. A family of chemokines attract leukocytes, whose persistence at an inflammatory site is important in the development of chronic disease [86].

Inflammation is referred to as a cancer “promoter”, because it induces cell proliferation, recruits inflammatory cells, increases cellular levels of ROS, thereby leading to oxidative DNA damage, and reduces DNA repair [86]. The deregulation of cell death and/or repair programs results in DNA replication and proliferation in chronically-inflamed tissue [86]. Inflammation also causes resistance to apoptosis, secretion of pro-angiogenic and immunosuppressive factors, invasion and metastasis [102]. All of these processes contribute to carcinogenesis. For example, the most common type of liver cancer results from chronic liver inflammation induced by either HBV or HCV [103,104]. The enhanced DNA replication and DNA damage created during chronic inflammation increase the number of free DNA ends in host genomic DNA and promote HBV integration [105]. In case of HCV, its core protein interacts with the signal transducer and activator of transcription 3 (STAT3) protein [106], a transcription factor involved in mediating cytokine signaling [107]. This interaction results in enhanced proliferation and upregulation of Bcl-XL and cyclin-D [106]. In this way, chronic liver inflammation induced by HCV may alter the local cytokine profile and the balance between apoptosis and proliferation.

### 4.2. Inflammation and DNA Damage induced by Co-Infections

Co-infections with certain sexually transmitted diseases (STD) cause cervical inflammation and increase the risk of cervical cancer in HPV-infected women [101,108,109]. Furthermore, high levels of inflammatory mediators, such as cyclooxygenase (COX)-2, an enzyme responsible for prostaglandin formation, are observed in cervical cancer [110,111]. However, this inflammation is not thought to be primarily due to the HPV infection itself, but rather due to other factors, such as co-infections. One reason for the lack of HPV-induced inflammation is that HPV infects basal keratinocytes that are distant from immune centers and have short lifespans. In addition, the virus does not destroy the cells it infects, thereby avoiding the triggering of inflammation [112]. Co-infections that do trigger inflammation can be of either viral or bacterial origin. For example, studies have determined that co-infection with either *Chlamydia Trachomatis* or HSV increase the risk of developing cervical cancer [113], as does infection with other STDs, such as *Neisseria gonorrhoeae* [114]. The inflammation induced by these co-infections can induce the generation of ROS, which can contribute to carcinogenesis by damaging DNA, as described below.

### 4.3. Oxidative Stressors, Viruses and Cancer

ROS and reactive nitrogen species (RNS) are charged free radicals that are primarily generated in mitochondria as by-products of aerobic respiration, cytochrome P450 activity and peroxisome function [115]. In normal conditions, the pro- and anti-oxidant systems maintain ROS homeostasis. A lack of proper balance between these two sets of systems results in changes to cellular levels of ROS and can lead to oxidative stress (OS). In general, the sources of OS can be divided into two broad categories: exogenous and endogenous. Endogenous OS, as discussed above, is primarily derived from natural processes, such as cellular signaling, metabolic processes and inflammation [116,117,118,119]. Exogenous and environmental sources include ionizing radiation, such as X-, γ- and cosmic rays, α-particles from radon decay, oxidizing chemicals and UVA solar radiation. For example, ionizing radiation generates radicals, including superoxide, hydrogen peroxide and hydroxyl radicals [120], most of which are generated during the radiolysis of water. Of these, the hydroxyl radical is the most damaging species and produces mostly single-strand breaks [121]. Overall, radiation induces genetic instability and chromosomal rearrangements, and many of these rearrangements are similar to those found in human cancers [122].

Chronic exposure to viral infections also induce the constant generation of free radicals [123], which can damage cellular biomolecules, including DNA. DNA damage produced by OS results in apurinic/apyrimidinic (abasic) DNA sites, oxidized purines and pyrimidines, and single- and double-stranded DNA breaks [124]. Therefore, the ROS- and RNS-induced oxidative and nitrative DNA damage that frequently occurs during inflammation can contribute to carcinogenesis [125,126].

A connection between OS, DNA damage and the incidence of hepatocellular carcinoma has been demonstrated by Hagen and colleagues [127]. The elevated level of ROS observed in chronic HBV-infected livers causes liver cell injury [127,128] and may lead to an accumulation of repeated genetic damage and an increased risk for genomic alterations in infected hepatocytes. Moreover, cell necrosis and proliferation in chronic HBV infection in response to cell injury could allow for greater exposure of DNA to ROS and incomplete repair of DNA damage. All of these factors are predicted to increase the probability of the fixation of genetic and chromosomal abnormalities, thus causing mutations and enhancing the development of HCC [127]. Among the proteins encoded by HBV, the X gene product (HBx) is a protein that increases virus gene expression and replication to maintain viral infection by transactivating cellular promoters and enhancers [129,130]. HBx induces OS and contributes to liver disease pathogenesis associated with HBV infection [131].

High levels of ROS can directly regulate NF-κB activation. This was demonstrated in EBV-positive Burkitt’s lymphoma cells, which have elevated ROS levels and altered NF-κB activation as opposed to EBV-negative Burkitt’s lymphoma cells [132]. EBV nuclear antigen-1 is the only viral protein expressed in all EBV-carrying malignancies, and it induces chromosomal aberrations and DNA DSBs through increasing ROS production. This effect can be reversed by antioxidants [133,134].

As opposed to several of these other viruses, infection with HPV does not in itself cause significant inflammation likely to lead to OS. However, expression of the smaller splice variant of E6, E6*, is able to increase ROS levels in oral keratinocytes. This ROS level increase is likely connected to the E6*-mediated decrease in the expression of the antioxidant enzymes, superoxide dismutase (SOD) 2 and glutathione peroxidase (GPx) 1/2 [135]. The increase in the level of oxidative stress due to E6* expression also resulted in an increase in DNA damage [135].

Other environmental and lifestyle-related factors also contribute to the induction of oxidative stress in infected cells. Among the factors connected to lifestyles that can promote virus-induced tumorigenesis are alcohol consumption and tobacco smoking. For example, heavy alcohol intake, defined as ingestion of more than 50–70 g/day for prolonged periods, is a well-established HCC risk factor [136]. Alcohol-induced oxidative stress plays an important role in the development of alcohol liver disease, and alcohol metabolism via the enzyme, alcohol dehydrogenase, results in increased ROS production, hepatocyte injury and apoptosis. Interestingly, all of these reactions could be blocked by the administration of antioxidants [137,138].

Smoking is strongly associated with an increased risk of developing cervical cancer in HPV-positive women [139,140]. Smoking is known to induce inflammation and oxidative stress [141], which may lead to DNA damage, integration and carcinogenesis, as discussed later.

Many studies have demonstrated that long-term use of oral contraceptives increases the risk of cervical cancer [142,143,144]. The strongest evidence for this connection comes from the large pooled analysis of the International Agency for Research on Cancer (IARC) studies for the role of oral contraceptive use in HPV-induced carcinogenesis. Although “ever use” of oral contraceptives was only moderately associated with cancer risk, a strong dose-response relationship with increasing years of use was observed [142].

High parity may also increase the risk of cervical cancer. One possible mechanism is that pregnancy maintains the transformation zone on the exocervix for many years [145], thereby facilitating direct exposure to HPV and other cofactors. Hormonal changes induced by pregnancy, such as increased levels of estrogen and progesterone, may also modulate the immune response to HPV along with viral persistence and/or progression [146,147]. High parity may also cause cervical trauma and cellular oxidative stress, thus leading to DNA damage and carcinogenesis [148]. In the IARC-pooled analysis, the odds ratio for cervical cancer in women with seven or more full-term pregnancies was four-fold higher than that in nulliparous women, and the risk increased linearly with an increasing number of full-term pregnancies [146].

## 5. Integration of Viral DNA into the Human Genome

**Figure 1:**
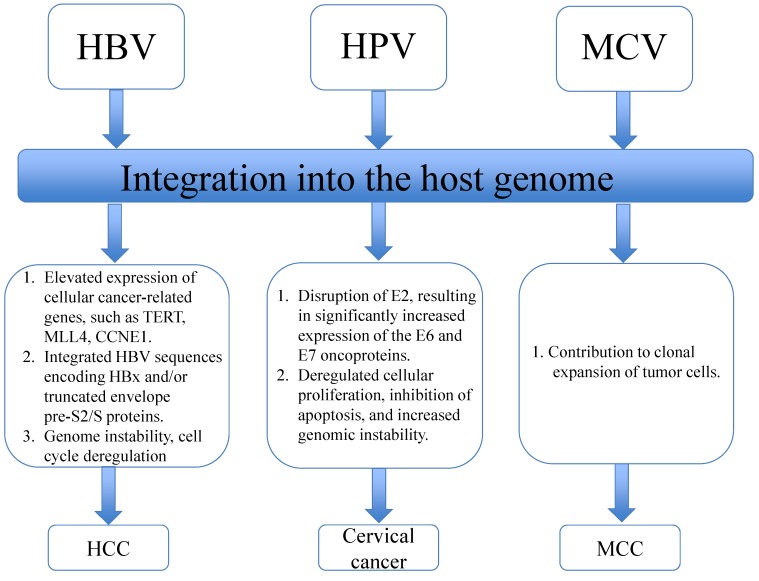
Integration of HBV, HPV and MCV viral DNAs into the human genome induces cellular and viral responses and further contributes to carcinogenesis (HBV, hepatitis B virus; HPV, high-risk human papillomaviruses; MCV, Merkel cell polyomavirus; MCC, Merkel cell carcinoma; TERT, telomerase reverse transcriptase; MLL4, Mixed-lineage leukemia 4).

Cancer incidence is associated with integration of the viral genome for several oncogenic viruses, including HBV, HPV and MCV. It is not yet clear that integration of EBV is a mechanism for carcinogenesis. The risk of virus integration depends on the level of DNA damage, because integration requires DSBs in both the host and virus DNA [149] (see Figure 1). Furthermore, a major cause of DNA damage is OS, which can be triggered by viruses and enhanced by exogenous factors.

### 5.1. Hepatitis B Virus (HBV)

HCC is the fifth most common cancer and the third leading cause of cancer death worldwide [136], and up to 80% of HCC is attributable to either HBV or HCV infection [150]. HBV is a partially double-stranded DNA hepadnavirus with retroviral features. The risk of HCC is increased five- to 15-fold in chronic HBV carriers compared with the general population [136], and studies have shown that integration of the HBV genome into the cellular genome is present in over 85%–90% of HBV-related HCCs. However, the integrated form of HBV is also present in non-tumor tissue of patients with chronic HBV infections. Integration of the HBV genome into hepatocytes occurs during persistent HBV infection and precedes development of HCC [151,152]. HBV integration leads to the elevated expression of several cellular cancer-related genes, such as TERT, mixed-lineage leukemia 4 (MLL4) (MLL4 is a part of the ASC-2 complex implicated in the p53 tumor suppressor pathway [153]) and CCNE1 (encoding cyclin E1) [154]. HBV integration is also associated with early onset of HCC and poor outcomes [154], and integrated HBV sequences encoding the HBx and/or truncated envelope pre-S2/S proteins are found in a large percentage of HCC [55]. Integration of the HBx sequence into host DNA in HCC promotes genetic instability through mechanisms that include the inactivation of the UV-damage DNA binding protein, so as to interfere with nucleotide excision repair, repression of p53-mediated gene transcription [155] and inactivation of p53-dependent apoptosis, cell cycle regulation, DNA repair and tumor suppression [156]. In addition, HBx transactivates several signaling pathways connected to carcinogenesis, including those mediated by protein kinase C, JAK/STAT and PI3K. [130,157]. HBx also upregulates TGF-β expression in HCC tissue and is thought to contribute to carcinogenesis through this mechanism, as well [158]. TGF-β is a cytokine that inhibits hepatocyte proliferation during liver regeneration [159,160,161] and stimulates extracellular matrix protein production by hepatocytes during liver cirrhosis [162,163]. Deletion of the preS2 region of the S2/S protein during HBV integration leads to a truncated envelope pre-S2/S protein that is frequently found in HCC samples [164] (see Figure 1). This truncated pre-S2/S product increases malignant transformation by transactivating several cellular genes, including c-myc, c-fos and c-Ha-ras [165,166]. Interestingly, the pre-S mutant large surface antigens can be retained in the endoplasmic reticulum and, thus, escape detection by the immune system. Moreover, this protein can initiate ER stress and thereby induce oxidative DNA damage and genomic instability [167]. The pre-S mutant also can upregulate COX-2 and cyclin A to induce cell-cycle progression and proliferation of hepatocytes [167]. Overall, the process of HBV integration induces additional genetic alterations, including chromosomal deletions, translocations, fusion of transcripts, amplification of DNA and generalized genomic instability [105,157], leading to overexpression of oncogenes, depletion of tumor suppressor genes and an altered microRNA profile [157].

The integration rate of HBV DNA into the host genome is significantly increased in the presence of DSBs [168]. If DNA damage is induced by the addition of H_2_O_2_, which increases cellular levels of ROS, the frequency of HBV integration also increases significantly [169]. Moreover, inhibiting poly(ADP-ribosyl)ation by adding the ADP-ribosylation inhibitor, 3-aminobenzamide, also leads to a significant increase in integration [169]. Poly(ADP-ribose) polymerase-1 (PARP-1) is an enzyme that is stimulated by DNA strand breaks caused by oxidative stress and other types of DNA damage and responds by carrying out poly(ADP-ribosyl)ation. Poly(ADP-ribosyl)ation is involved in DNA damage repair processes, such as base excision repair and suppression of genetic recombination [170,171,172,173].

Integration of HBV into the human genome can occur near or within fragile sites in genes that regulate cell signaling, proliferation and viability [174]. Common targets of integration include genes for human cyclin A2 [175], the PDGF receptor, calcium signaling-related genes, mixed lineage leukemia encoding genes, 60S ribosomal protein genes [174], human telomerase reverse transcriptase (hTERT) [176] and the retinoic acid receptor β [177]. The data produced by next-generation sequencing (NGS) now allows researchers to not only determine the sites of integration, but also to identify the mutations and to specify DNA damage types that contribute to carcinogenesis [178,179]. NGS studies have shown that HBV integrations into TERT, MLL4, CCNE1 and ANGPT1 (encoding angiopoietin 1) [154,180,181] can and do occur. NGS has also identified frequent mutations in CTNNB1 (encoding β-catenin), IRF2 (encoding interferon regulatory factor 2), TP53, ARID2 (subunit of the polybromo- and BRG1-associated factor chromatin remodeling complex [182]; functioning as a tumor suppressor gene [183]) and ARID1A (encoding a component of the SWI/SNF chromatin remodeling complex) [183,184,185]. These mutations can be associated with disease etiology. For example, ARID2 mutations are significantly enriched in HCV-associated HCC [183], and ARID1A may be crucial in HCC invasion and metastasis [185]. IRF2 mutations have been associated with hyperploidy and high chromosomal instability [184], while TP53 inactivating mutations result in an altered p53 pathway [184]. Interestingly, CTNNB1 mutations may define a homogenous subtype of HCC not related to HBV infection [184].

### 5.2. Human Papillomaviruses (HPVs)

HPVs are a group of circular, double-stranded DNA viruses that infect epithelial cells. More than 100 different genotypes of HPV [186] have been described; of these, a subset infects the anogenital area, and within this subset, the individual types are classified as either high risk or low risk. High-risk HPVs can cause cancerous lesions, while low-risk HPVs do not [187]. An important difference between high- and low-risk HPVs is that high-risk HPVs show a greater tendency to integrate into the host genome, thereby causing high-grade lesions and cancer, while low-risk types are preferentially maintained as extrachromosomal circular episomes [188,189].

HPV is known to cause virtually all cases of cervical cancer, which is the second most common cancer in women worldwide and the fourth most common cause of cancer death in women worldwide [190,191]. More than 270,000 women die from cervical cancer each year, and according to 2013 data from the WHO, the developing world accounts for more than 85% of these cases. More than 90% of premalignant and malignant squamous lesions in the uterine cervix are HPV DNA positive [192,193]. Moreover, HPV16, HPV18, HPV31 and HPV33 account for 90% of all cases of cervical cancer. Among these high-risk HPVs, HPV type 16 is the most prevalent type and by itself accounts for more than 50% of all cases of cervical cancer [194,195]. High-risk HPV infection is also associated with several other anogenital and oropharyngeal cancers. For example, it is thought to be responsible for more than 90% of anal cancers, 70% of vaginal and vulvar cancers, 60% of penile cancers and 63% of oropharyngeal cancers [196].

The majority of HPV infections are spontaneously cleared by human cells [197,198]. Cervical intraepithelial neoplasia (CIN) 1 lesions develop occasionally, but most of these lesions will be spontaneously cleared and fail to progress [199]. However, some HPV-infected women can be co-infected by other viruses or bacteria, then develop cervical inflammation, as noted above. The cellular proliferative and anti-apoptotic effects of inflammation, combined with low-level expression of the E6 and E7 oncogenes encoded by the episomal HPV, contribute to CIN1 to CIN2 progression [101]. At the CIN2 stage, the generation of ROS and RNS may create DSBs in both the viral and host DNA. This can then allow HPV to integrate into the human genome, thereby enabling overexpression of the E6 and E7 oncogenes, which then facilitate the transition to CIN3 and, sometimes, invasive carcinoma.

The oncogenes encoded by HPV play crucial roles in carcinogenesis. Typically, the levels of E6 and E7 oncogene expression from episomal HPV16 are low. *In vitro*, high-level expression of HPV oncogenes from integrated HPV forms is generally preceded by the loss of episomal HPV [200,201]. HPV-16 integration often disrupts the E2 gene (the E2 ORF has been identified as a preferential site of integration), resulting in significantly increased expression of the E6 and E7 oncoproteins due to loss of negative feedback control by the viral regulatory E2 protein [149,202] (see Figure 1). Consistent with this model, transcriptional activity of integrated HPV-16 DNA can be suppressed by E2 proteins from the episomal form [149,200,203,204,205].

Elevated expression of HPV oncogenes can also be achieved by the presence of an increased number of episomal HPV-16 copies. A high episomal HPV-16 load, combined with an absence of HPV-16 integration, is frequently observed in CIN, while a high proportion of invasive cancers contain integrated HPV-16 forms, suggesting that integration is an important factor in progression from high-grade lesions to invasive cancer and may be a potential marker for CIN progression risk assessment [206,207].

Accumulating evidence suggests that among those factors that can increase the risk of HPV integration are those factors that generate excessive amounts of ROS and RNS. Nitrative and oxidative DNA damage were found in high-risk HPV infections, especially nitric oxide synthase-dependent DNA damage, which is believed to play a critical role in inflammation-mediated cervical carcinogenesis [208]. Exposure to high and sustained levels of nitric oxide (NO) will increase DNA damage and mutation frequencies, supporting the idea that NO is a cofactor with infection in cervical carcinogenesis [209]. Furthermore, ROS and RNS have the potential to create DNA strand breaks [209,210], thereby increasing HPV-DNA integration into cellular chromatin. As discussed above, these factors may be virus mediated (*i.e.*, E6* expression) [135], mediated through infection or co-infection (inflammation) or exogenously derived [101]. Our laboratory has demonstrated that chronic oxidative stress induced by E6* expression is able to increase DNA damage [135], and it has also been shown by other laboratories that the frequency with which foreign DNA constructs containing antibiotic resistance markers integrate into genomic DNA is increased by the induction of DSBs, either by HPV16 E6 and E7 expression or by spontaneous breakage at fragile sites in specific cancer cell lines [211,212,213]. Therefore, the rate of HPV integration seems to correlate with the level of DNA damage.

A number of pathways are affected by E6 and E7 expression [214]. As mentioned earlier, E6 mediates the rapid degradation of p53 and activates hTERT, while E7 inactivates pRB. In addition, E6 binds to IRF-3 and inhibits its transcriptional activity, which may provide HPV with the ability to circumvent the normal antiviral response [215]. E7 sequences mediate the activation of cyclin E, followed by the activation of cyclin A, which is required for transformation [216]. In addition, the cooperative interactions between the E6 and E7 proteins leads to cellular immortalization [217,218], likely through a combination of specific mechanisms, such as those noted above. In summary, the elevated expression of oncoproteins from integrated forms of HPV deregulates cellular proliferation, blocks apoptosis and increases genomic instability, all of which contribute to cellular transformation (see Figure 1).

HPV integration sites are distributed randomly throughout the host genome, without a single region predominating [219]. However, 38% of 192 integrants were found in known common fragile sites (CFSs), and there was no evidence to suggest targeted disruption or functional alteration of critical cellular genes by the integrated viral sequences [219]. However, some studies have demonstrated that high-risk HPV integration has occurred within or adjacent to known oncogenes, most commonly within intronic sequences [219,220,221,222]. For example, the region of the MYC gene at chromosomal band 8q24 is a frequently observed integration site in HPV18-positive cervical cancer [221,222,223]. Recently developed NGS-based methods now provide a very efficient method to map HPV integration sites. One NGS study, for example, showed that the 3'-breakpoints of integrated HPV16 DNA distribute preferentially within the early region E1-PAE segment in HPV 16. This indicates the importance of deregulated viral oncogene expression for carcinogenesis [224]. Interestingly, about half of the mapped HPV16 integration sites directly target human cellular genes [224]. These studies suggest that the insertional mutagenesis of the host genome may play a role in at least some cervical cancers [149]. However, many publications using NGS focus on identifying genotype and determining HPV load, rather than on identifying sites of integration [225,226]. Further studies are needed to identify integration sites, genomic mutation and DNA damage.

### 5.3. Merkel Cell Polyomavirus (MCV)

MCV is a double-stranded DNA polyomavirus, shown to be associated with Merkel cell carcinoma (MCC) through the use of NGS in 2008 [29]. These tumors display MCV DNA in an integrated form within the tumor cell genomes in a clonal pattern, suggesting that MCV infection and integration contribute to clonal expansion of the tumor cells [29,227] (see Figure 1). The MCV genome encodes multiple splice variants of a tumor (T) antigen protein complex that targets several tumor suppressor proteins, such as pRB and p53 [228]. One of these splice variants, the large tumor antigen, is mutated in MCV-positive MCC tumors cells, and this selective mutation affects the cellular DNA damage response to prevent auto-activation of integrated virus replication, disrupt host genomic integrity and inhibit cellular proliferation [228,229,230]. Several features of this virus, such as the frequent and selective association of MCC with MCV, integration of MCV into the host genome, the recurrent pattern of conserved viral DNA sequences and the constant expression of viral oncoproteins are very similar to those seen in high-risk HPV-induced cervical cancer [55]. However, the exact role of integration in MCC carcinogenesis requires further study.

### 5.4. Epstein-Barr Virus (EBV)

EBV is a double-stranded DNA herpesvirus that is primarily associated with Burkitt’s lymphoma, nasopharyngeal carcinoma and several lymphoproliferative disorders [30]. Burkitt’s lymphoma appears in three main clinical variants—the endemic, sporadic and immunodeficiency-associated variants. EBV is detected in 96% of cases of endemic variant Burkitt’s lymphoma involving the jaw, which is the most common malignancy of children in certain areas of central Africa [231]. In contrast, EBV is rarely associated with the sporadic variant of Burkitt’s lymphoma, and the jaw is less commonly involved [232]. EBV-associated Burkitt’s lymphoma is common in individuals lacking efficient T-cell function, such as AIDS patients or transplant recipients [233].

All Burkitt’s lymphomas have chromosomal translocations that place the MYC oncogene under the control of the Ig heavy chain or one of the light-chain loci, which induce MYC deregulation and contribute to the pathogenesis associated with Burkitt’s lymphoma [234,235,236]. EBV also displays transformative abilities, as EBV-encoded latent genes induce B-cell transformation into permanent, latently-infected lymphoblastoid cell lines (LCLs) *in vitro* by altering cellular gene transcription and activating important cell-signaling pathways [237].

EBV usually persists in an episomal state with multiple copies of circular DNA. Integration of the EBV genome into that of the host is rare and is unlikely to contribute to most cases of Burkitt’s lymphoma. Such integration has been only shown in several Burkitt’s lymphoma cell lines, such as IB4, BL-36, BL-60 and BL-137 [238,239,240,241,242]. Integrated, episomal and linear copies of EBV DNA can coexist in Burkitt’s lymphoma cells [241]. EBV integration into fragile sites of the host chromosome is associated with partial deletion of the viral genome and generates a region of enhanced chromatin instability in the host cell genome [238]. This genome instability can induce the loss of host genes, such as BACH2, which is a putative tumor suppressor gene, and this may contribute to lymphomagenesis [243].

In addition to Burkitt’s lymphoma, EBV is also associated with nasopharyngeal carcinoma (NPC). The undifferentiated form of NPC, classified by WHO as type III, shows the most consistent association with EBV worldwide, especially in particular areas of China and south-east Asia [244]. An association between EBV and WHO types I and II NPC has also been demonstrated [245]. Evidence of the importance of EBV integration for the development NPC is not conclusive, and integration of EBV was demonstrated in only a subset of studies. For example, integrated EBV was found in four of 17 pathologically-diagnosed, EBV-positive NPC biopsy samples [246]. Another study showed that EBV DNA was integrated into chromosomes in the EBV-positive NPC cell lines, HSB4 and H2B17-7. However, the exact role of integration of EBV in NPC is not clear.

## 6. Prevention and Reduction of Risk Factors for Cancers

Knowledge of the etiology of virus-mediated carcinogenesis, the networking of pathways involved in the transition from infection to cancer and the risk factors associated with each type of cancer, all suggest prophylactic and therapeutic strategies that may reduce the risk of virus-mediated cancer. Prophylactic vaccination provides one such strategy; HPV vaccines can effectively reduce the incidence of HPV infections and are anticipated to correspondingly reduce the incidence of HPV-associated cancers and pre-cancerous lesions over the next several decades [247]. As discussed in this current review, lifestyle choices also matter, and education on the risk factors associated with these lifestyle choices can be considered a prophylactic measure targeting cancer prevention. We also discussed factors that contribute to virus-mediated carcinogenesis, including integration of the viral genomes of HBV, HPV and MCV. Typically, cancer develops years to decades following the initial infection with HBV and HPV. This delay provides us with a unique opportunity for cancer interception, especially in cases where the tumorigenesis process requires virus integration. In these situations, approaches that would decrease the probability of integration are anticipated to reduce the number of cancer cases. Theoretically, therapeutic or dietary measures directed against oxidative stress could reduce oxidative stress in already infected cells [248] and thereby diminish the risk of viral integration. Dietary antioxidants can, in some cases, supplement the activity of endogenous antioxidants found in normal cells and fortify them against challenges posed by increased levels of ROS. In the case of cervical cancer, studies have shown that several antioxidants, such as reduced glutathione, GPx, glutathione-S-transferase, SOD and antioxidant vitamins (vitamin E, vitamin C, lutein, beta-carotene, lycopene and zeaxanthin), are reduced in the circulation of cervical cancer patients [249,250]. In contrast, the concentration of the lipid peroxidation parameter, malondialdehyde, was significantly higher in women with CIN or invasive cervical cancer [250]. That may be due to the increased utilization of the antioxidants to scavenge oxidative stress-induced lipid peroxidation and their sequestration by tumor cells. A deficiency in antioxidant vitamins may, therefore, contribute to the increased prevalence of cervical cancer observed in women with a low socioeconomic status [249]. Possible benefits from dietary antioxidant consumption, a highly researched topic for the last two decades, still remain debatable [251,252]. In addition, clinical trials addressing the effect of antioxidant therapies on cancer have not yet provided a clear answer [253]. Future development of therapies for the reduction of cellular oxidative stress must take into account the complexity of problems inherent in the regulation of redox balance.

## 7. Conclusions

Viruses have been demonstrated to be the causative agents of approximately 10%–15% of all cancers worldwide. Viruses, when acting as carcinogenic agents, utilize a variety of mechanisms to transform human cells. Their genome encodes proteins that reprogram the normal functioning of cells so as to favor virus production, and the most common outcome for this virus-induced reprogramming is the induction of genomic instability, as seen by the accumulation of point mutations, aberrations and DNA damage. However, the mechanisms causing this genomic instability differ between viruses. Genomic instability caused by viruses serves as a major step on the pathway leading to carcinogenesis and can be induced by viral infection and inflammation, by viral gene expression and by exogenously derived factors. All of these mechanisms can lead to increased oxidative stress, which is then able to damage host DNA. For some viruses, such as HBV, HPV and MCV, the pivotal step of the onset of tumorigenesis appears to be integration of the viral genome, which elevates viral oncogene expression and promotes cancer progression. Typically, cancer develops years to decades following the initial infection. This provides us with a unique opportunity for cancer interception, especially in cases where the tumorigenesis process requires virus integration. Our increasing understanding of the etiology of virus-mediated carcinogenesis, the networking of pathways involved in the transition from infection to cancer and information on risk factors specific for each type of cancer will continue to suggest prophylactic and therapeutic strategies to reduce the risk of virus-mediated cancer. However, the detailed mechanisms of how integration causes and accelerates carcinogenesis are still not fully understood and require further study.
